# Correction: Effects of using standing desks for 45 minutes on the stress and executive function of elementary school students

**DOI:** 10.1371/journal.pone.0342737

**Published:** 2026-02-10

**Authors:** The PLOS One Staff

## Notice of Republication

This article was republished on January 26, 2026, to correct errors in tables 2–4. The publisher apologizes for the errors. Please download this article again to view the correct version. The originally published, uncorrected article and the republished, corrected articles are provided here for reference.

## Supporting information

S1 File Originally published, uncorrected article. (PDF)

S2 File Republished, corrected article. (PDF)
