# Supplementary material for: Correction: Effects of using standing desks for 45 minutes on the stress and executive function of elementary school students
Source: PLoS One. 2026 Feb 10;21(2):e0342737. doi: 10.1371/journal.pone.0342737 (PMC12890118; doi:10.1371/journal.pone.0342737)
Supplement: S1 File — (PDF) [file pone.0342737.s001.pdf]

PLOS ONE

RESEARCH ARTICLE

Effects of using standing desks for 45 minutes on the stress and executive function of elementary school students

Ryo Tanaka[1,2]*, **Shingo Noi**[2]

1 School of Physical Education, Osaka University of Health and Sport Sciences, Sennan-gun, Osaka, Japan, **2** Research Institute for Health and Sport Science, Nippon Sport Science University, Setagayaku, Tokyo, Japan

* r-tanaka@ouhs.ac.jp

Check for updates

OPEN ACCESS

Citation: Tanaka R, Noi S (2022) Effects of using standing desks for 45 minutes on the stress and executive function of elementary school students. PLoS ONE 17(8): e0272035. https://doi.org/10.1371/journal.pone.0272035

Editor: Cosme F. Buzzachera, University of Pavia: Universita degli Studi di Pavia, ITALY

Received: December 20, 2020

Accepted: July 12, 2022

Published: August 18, 2022

Copyright: © 2022 Tanaka, Noi. This is an open access article distributed under the terms of the Creative Commons Attribution License, which permits unrestricted use, distribution, and reproduction in any medium, provided the original author and source are credited.

Data Availability Statement: Our data contain potentially identifying information and ethics committee of Nippon Sport Science University does not allow us to upload the data publicly. The datasets analysed in this study are available from the individuals that accept queries regarding data access. Nippon Sport Science University. Address: 7-1-1 Fukasawa, Setagayaku Tokyo 158-8508, Japan. E-mail: shikano.a@nittai.ac.jp

Funding: This research was conducted as part of collaborative research with Okamura Corporation. The height-adjustable standing desks and research

Abstract

Studies have presented data regarding the effects of short-term (weeks) and long-term (one year) use of standing desks in classrooms on children's health, cognition, and musculoskeletal symptoms. However, no previous study has examined such effects in an extremely short-term period such as one classroom lesson. This study aimed to examine the effects of using standing desks for 45 minutes on elementary school students' stress and executive function. For this experiment, 56 students were recruited from public elementary schools in Setagayaku, Tokyo, Japan. There were three conditions involving the use of standing desks (standing, sitting, and mixed), and all students performed tasks for 45 minutes in each condition. Measurements of stress and executive function were performed before and after the students engaged with each condition. Stress levels did not differ between the sitting and standing conditions for the full 45 minutes. The number of correct answers in the Stroop test, an interference task, was higher in the standing and mixed conditions (switching between standing and sitting) than in the sitting condition (interaction: $F_{(1,37)} = 3.340$, $p = 0.04$, $\eta^2 = 0.05$). These results indicate that using standing desks for 45 minutes improved the 'inhibition' of executive function without excessively increasing stress levels.

Introduction

It is well known that sedentary behaviour (sitting, lying, reclining, and expending ≤ 1.5 metabolic equivalents) has an adverse influence on health [1–3]. In children, it has been frequently reported that excessive sedentary behaviour relates to physical and/or mental impairment [4–6]. Movement is important for healthy growth and development [3], and schools are generally tasked with promoting children's health; however, in classrooms, where children spend a great deal of their waking hours, students are seated 50–70% of the time [7–10]. Nevertheless, physical activity at school can be increased by changing the environment [11].

Changing traditional classrooms into environments that encourage active behaviours can help ensure that students move regularly during lessons. Previous studies have sought to identify methods of increasing movement and reducing sedentary behaviour in classrooms by

funds were provided by Okamura Corporation. This study was supported by grants from the Grants-in Aid for Scientific Research (no. 18J15510) from the Japan Society for the Promotion of Science fund. The funders had no role in study design, data collection and analysis, decision to publish, or preparation of the manuscript.

Competing interests: The height-adjustable standing desks used in the present study were supplied via an in-kind donation from Okamura Corporation. The company played no role in study design, data collection and analysis, decision to publish, or preparation of the manuscript. There are no patents, products in development or other marketed products to declare. This does not alter our adherence to PLOS ONE policies on sharing data and materials.

determining the efficacy of replacing traditional desks with standing desks [12]. Although another study found no increase in physical activity with introduction of standing desks [13], such studies on sedentary behaviour have involved before-and-after comparisons of associated physical activity, with examination periods ranging from weeks to months [14]. One study reported that the introduction of height-adjustable standing desks decreased sitting time during school time, and no compensatory increases in sedentary behaviour occurred during non-school time in the intervention class [15]. Meanwhile, another study reported that this measure improved executive function related to academic performance [16]. In addition, the introduction of standing desks in the classroom for three weeks or three months has been found to have no adverse effects on musculoskeletal symptoms and sleep quality [17–19]. Some researchers have suggested that the observed benefits of standing desks are solely attributable to the novelty of using these desks; however, Parry et al. [20] indicated that the effects outlast any sense of novelty. Parry et al. [20] also reported that using a standing desk for the entire school year is, along with increased standing time and reduced sitting time, less likely to induce discomfort in the neck and shoulders.

The current body of literature regarding this topic includes data regarding the short-term [17], mid-term [19], and long-term [20] effects of using standing desks in classrooms on physical activity, executive function, musculoskeletal symptoms, and sleep. However, despite these findings, standing desks remain largely absent from schools. Investigations have been conducted for periods of weeks, months, and one year among elementary school students, but to date no studies have examined the effects of using standing desks for one class period. Teachers want not only to improve children's potential (e.g., academic performance and executive function), but also to appropriately manage their classes [21]. Many teachers are not interested in how the introduction of standing desks will impact children in the distant future but are interested in how it will impact those who will participate in the class tomorrow. Demonstrating that the use of a standing desk for an extremely short-term period would not adversely affect stress, but might improve executive function, which refers to the group of cognitive processes that guide human behaviour, would be an important finding for teachers. The current study aimed to examine the effects on stress and executive function of elementary school students using standing desks for 45 minutes.

Materials and methods

Study design and equipment

This study was conducted in the laboratory between July and August in both 2017 and 2018 during school summer vacation, using a repeated-measures within-subject counter-balanced design. The height-adjustable standing desk used in this study was the 'Stafit' (Okamura Corporation Japan; Fig 1). This standing desk could be easily fixed at any height between 72.5 cm and 102.5 cm. A chair without adjustable height was used with the desk. Each participant was assigned a standing desk, and three conditions regarding the use of the standing desks were introduced, each comprising a 45-minute period. The first involved working on a task while standing for the full 45 minutes (standing condition); the second involved working on a task while sitting for the full 45 minutes (sitting condition); and the third involved working on a task while switching between standing and sitting every 10 minutes (mixed condition). In this latter condition, participants could freely choose to stand or sit for the final five minutes of the 45-minute session. The participants performed the experiment over three non-consecutive days, participating in a different condition each day. In all conditions, the task performed by the children constituted completing their summer vacation homework. In Japan, workbook is often provided by the school as a summer vacation homework, which includes answering

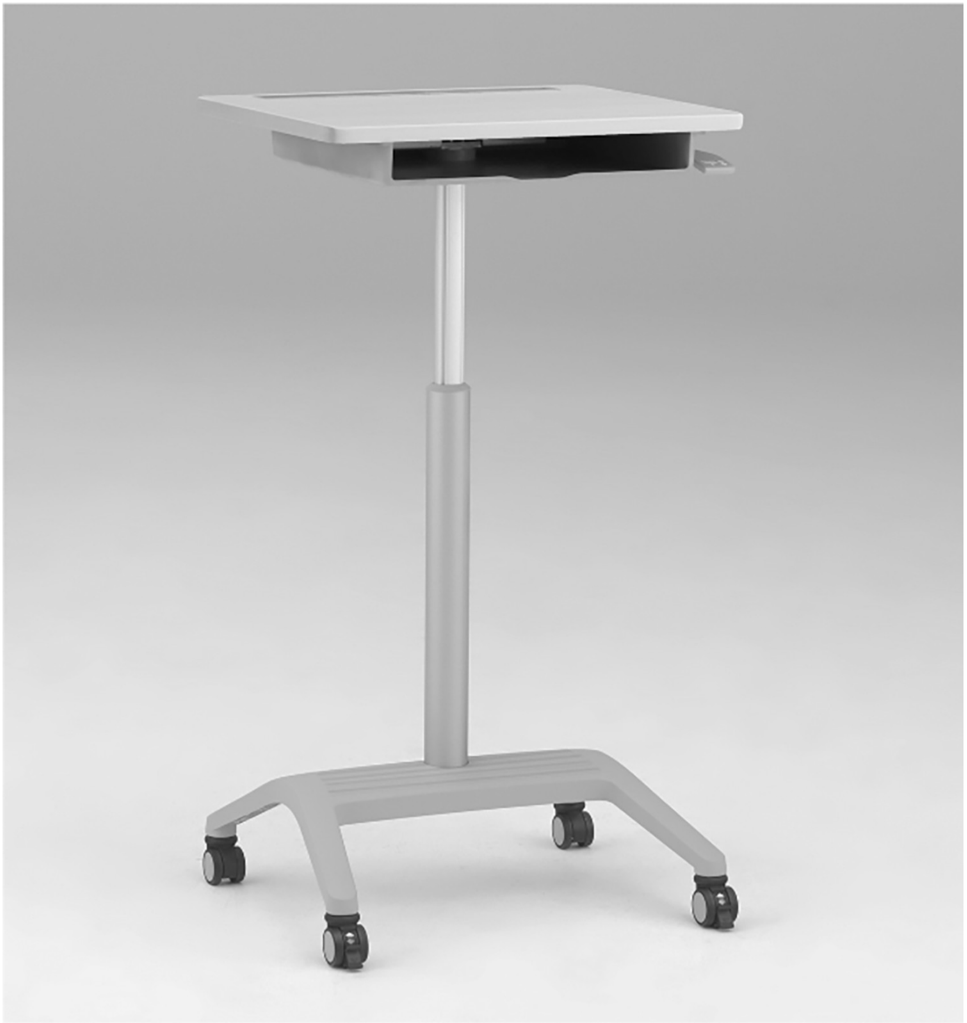

Fig 1. Example of the standing desk used in this research.

https://doi.org/10.1371/journal.pone.0272035.g001

questions about science and social studies, writing Chinese characters repeatedly, and solving numerical calculation drills. The drills are organised in booklets and handouts. The homework must be submitted to the homeroom teacher at the end of summer vacation. Most participants performed drill-based homework involving mathematics or language. The homework subjects differed between participants; however, since the participants attended public schools in the same district, no significant differences in the format or difficulty of the homework were found. No participants worked on creative activities such as painting. Measurements of stress and executive function were performed right before and after each condition (Fig 2).

Participants

Participants were recruited from public elementary schools (4th–6th grades, 9–12 years old) in Setagayaku, Tokyo, Japan, using flyers describing the outline of this study. For the 2017 research, the participant sample comprised 26 children. Here, data for 18 children (ten boys and eight girls) were analysed, as two children who could not participate in all elements of the research and six children who provided missing data were excluded. For the 2018 research, the

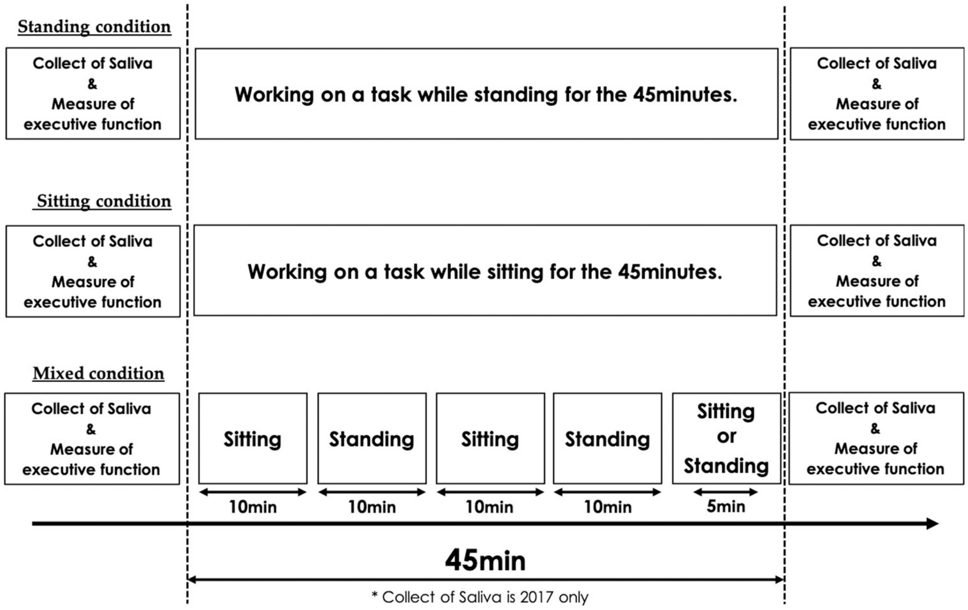

Fig 2. Experimental procedure of this study.

https://doi.org/10.1371/journal.pone.0272035.g002

participant sample comprised 30 children. Here, data for 25 children (14 boys and 11 girls) were analysed, as three children who could not participate in all elements of the research and two children who provided missing data were excluded. All participants were healthy, without any specified illness or disability. Of these, ten participated in both the 2017 and 2018 research, with data from 5 of the 10 participants included within this analysis. This study was conducted in accordance with the Declaration of Helsinki, and the Ethics Review Committee of Nippon Sport Science University (approval no. 017 –H50) approved the study protocol; further, the study was conducted after the students' parents provided written informed consent. In addition, before commencing the study, we explained to the students and their parents the purpose and contents of the research, that participation was voluntary and could be discontinued at any time, and that their privacy would be protected. Accordingly, the parents confirmed their consent by submitting signed consent forms.

Measurements

Stress. There are two systems that elevate substances in saliva in response to physical and mental stress: the sympathetic nervous-adrenal medullary system and the hypothalamic-pituitary-adrenocortical system [22]. The sympathetic nervous-adrenal medullary response increases concentrations of amylase and chromogranin A, and the hypothalamic-pituitary-adrenocortical system response increases concentrations of cortisol [22]. All these markers are responsive to acute stress. In this study, concentrations of amylase, chromogranin A (to measure the nervous-adrenal medullary response), and cortisol (to measure hypothalamic-pituitary-adrenocortical system response) were measured in the participants' saliva before and after they performed the 45-minute tasks. Saliva was collected using a saliva sampler (Salivette®, Sarstedt Ltd., Nümbrecht, Germany), for which participants were instructed to masticate specialised cotton for 30 seconds to one minute. Analysis of the saliva was performed by a professional vendor (SRL Co., Japan). These measurements were conducted only on the 2017 sample.

Executive function. Executive function comprises three sub-elements: 'inhibition', 'shifting', and 'updating' [23]. In this research, each sub-element was measured as follows:

'Inhibition' was measured using a Stroop test. A Stroop test composed of two tasks (a non-interference task and an interference task) was used. In the non-interference task, the participants were presented with a square that was coloured red, blue, yellow, green, or black, and were shown a list of these five colour names, all of which were written in black-coloured text. Participants were asked to select from the list the colour name that corresponded to the colour shown. In the interference task, participants were presented with a colour name written in red-, blue-, yellow-, green-, or black-coloured text, and were shown a list of the five colour names, again written in black-coloured text. Participants were asked to select the colour name from the list that corresponded to the colour of the text. In this task, the colour name and the colour in which it was written did not match (e.g. the word 'red' was written in blue font). For both tasks, participants were given a 10-second practice period and then performed the tasks over a 60-second period. The number of correct answers was then measured.

'Shifting' was measured using a trail-making test. The trail-making test was composed of two tasks. Task A involved connecting the numbers 1 to 25 in a solid line. Task B involved alternately connecting numbers 1 to 13, as well as 12 hiragana characters, one of the syllabaries used in the Japanese written language (e.g. number -> hiragana -> number -> hiragana, and so on). The 12 characters comprised the first characters that Japanese children learn ('あ' to 'し'). In this research, the total time taken to complete both tasks was measured, and the ratio between the two tasks was calculated (i.e., the ratio of the total time taken to complete task B to the total time taken to complete task A). Participants were instructed to connect the numbers and hiragana characters as quickly as possible.

'Updating' was measured using a reading-span task. This task was conducted with reference to the group reading-span task administered to elementary school students by Higuchi et al. [24]. Participants were instructed to read aloud short sentences displayed on a screen and, while reading, to memorise words underlined in red. Once they had finished reading all sentences, they were asked to report the underlined words. Conditions in which two, three, and four short sentences, respectively, were read aloud were introduced, and in each condition, the students repeated the task three times. For each condition, one point was given when all words were reported in the correct order. The total score was calculated by summing up the scores for each condition [24]. Subjects, verbs, adverbs and adjectives, onomatopoeia, and mimetic words were included in the sentences. There was no relationship among the conditions regarding the sentences and the words underlined in red. There was no bias regarding the type of words to be memorised.

Among these measures of executive function, the Stroop test was conducted in 2017 and 2018, and the trail-making test and the reading-span task were conducted in 2018 only.

Data analysis. The normality of the distributions of all measured values regarding stress and executive function (both before and after engagement in each condition) was assessed using the Shapiro-Wilk test. The concentration of amylase, chromogranin A, and cortisol in saliva and the number of correct answers in the Stroop test (both the non-interference task and the interference task) were confirmed to be normally distributed; however, the other measured values were not. A repeated two-way analysis of variance (ANOVA) was used to analyse the concentration of amylase, chromogranin A, and cortisol in saliva and the number of correct answers in the Stroop test. Meanwhile, the Wilcoxon rank sum test was used to analyse the other measured values. Statistical analyses were conducted using SPSS version 25 (SPSS, Inc., IBM, Armonk, NY, USA). Differences were considered to be statistically significant when $p < 0.05$, and where appropriate effect sizes were reported.

Table 1. Data of the participants used in the analysis of this study.

Year of Measurement	Measurements		Participants in 2017 only	Participants in 2017 and 2018	Participants in 2018 only
2017	Stress	Amylase	○	○	
		Chromogranin A	○	○	
		Cortisol	○	○	
	Executive function	ST	○		
2018	Executive function	ST		○	○
		TMT		○	○
		RST		○	○

ST: stroop test; TMT: trail-making task; RST: reading-span test.

https://doi.org/10.1371/journal.pone.0272035.t001

For participants who participated in both data collection periods (2017 and 2018), there were two data sets representing the number of correct answers for the Stroop test (both the non-interference and the interference tasks). For these participants' data, considering the potential influences of performing the other tasks to assess executive function, we only analysed the data for the 2018 research, during which the participants had also been examined concerning other sub-elements of executive functions ('shifting' and 'updating'; Table 1).

Results

First, we examined the effect of using standing desks for 45 minutes on the participants' stress levels. For all conditions, the concentration of cortisol in saliva was significantly higher after performing the task, $F_{(1,13)} = 32.449$, $p = 0.00$, $\eta^2 = 0.35$; however, the interaction was not significant (Fig 3). Examination of the concentration of amylase and chromogranin A in saliva showed that the main effects and interaction were not significant (Fig 3).

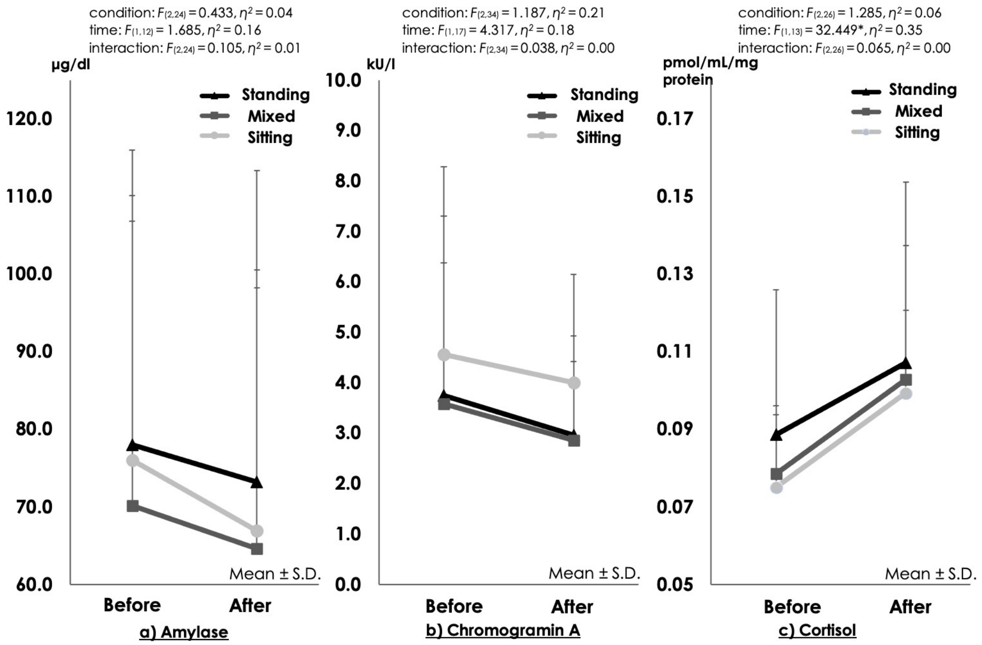

Fig 3. Comparison, for each condition, of the changes in stress hormones in saliva before and after performing the 45-minute tasks (n = 18).

https://doi.org/10.1371/journal.pone.0272035.g003

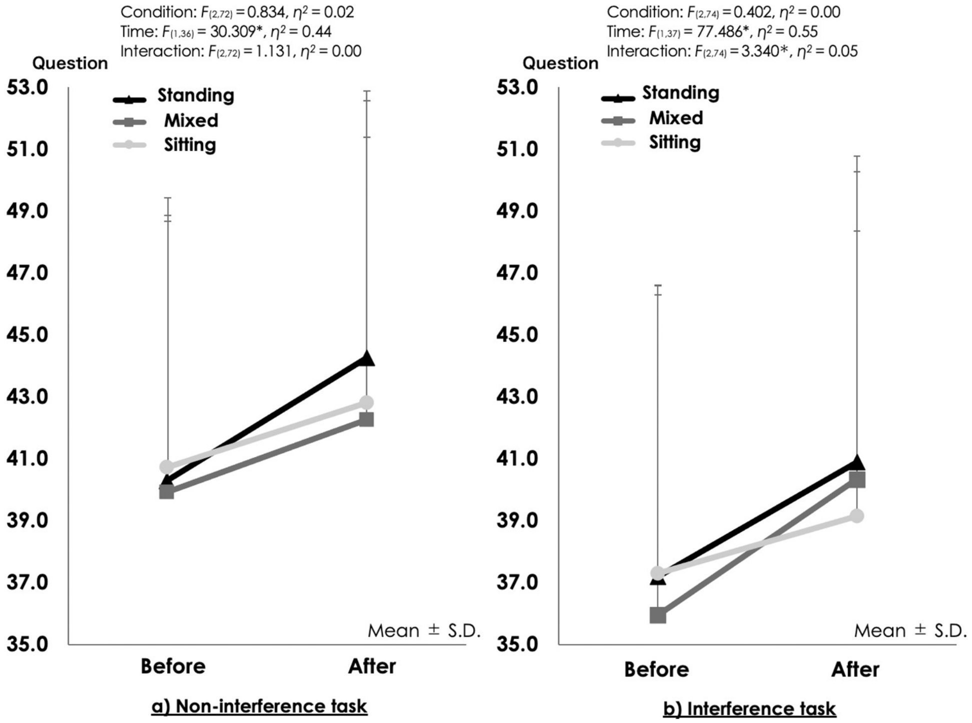

Fig 4. Comparison, for each condition, of the number of correct answers for the Stroop test before and after performing the 45-minute tasks (n = 38). Data from 2017 from the five participants who participated in both data collection periods (2017 and 2018) were not included.

https://doi.org/10.1371/journal.pone.0272035.g004

Second, we examined the effect of using standing desks for 45 minutes on the participants' executive function. Comparing the number of correct answers in the Stroop test (both the non-interference task and the interference task) by condition revealed that although the number of correct answers in the non-interference task significantly increased after the task, $F_{(1,36)} = 30.309$, $p = 0.00$, $\eta^2 = 0.44$, the interaction was not significant (Fig 4). For the number of correct answers in the interference task, there were significant interaction effects, with higher values for the standing (37.2 ± 9.4 times to 40.9 ± 9.9 times) and mixed conditions (36.0 ± 10.4 times to 40.3 ± 9.9 times) as compared to the sitting condition, $F_{(1,37)} = 3.340$, $p = 0.04$, $\eta^2 = 0.05$; (Fig 4).

Total times for the trail-making test (task A and task B), the ratio for the total times (task A to task B), and the total score for the reading-span task were analysed using the Wilcoxon rank sum test. This showed that the total time for task A in the standing and mixed conditions and the total time for task B in the standing condition significantly decreased but that the change in the ratio of the total times and the total score for the reading-span task did not significantly differ across the conditions (Tables 2–4).

Discussion

This study examined the effects of using standing desks for 45 minutes on children's stress levels and executive function. For all conditions, the main effect of time was only significant for the concentration of cortisol in saliva; for all stress-related values, the interaction effect was not significant (Fig 3). These results indicate that none of the conditions affected stress differently. Although some teachers believe standing desks cause children to become distracted and the

Table 2. Values for executive function before and after performing the standing condition task.

		Before[a]	After[a]	Wilcoxon sum rank (z)[b]	Effect size (r)
TMT	Total time to complete task A (sec)	28.0 (23.5–32.0)	24.0 (20.5–28.0)	-2.78*	0.56
	Total time to complete task B (sec)	58.0 (45.5–71.0)	46.0 (42.0–61.5)	-3.14*	0.63
	Ratio for total times (BA)	2.1 (1.8–2.4)	2.1 (1.7–2.3)	-1.22*	0.24
RST	Total score of reading span task	4.0 (3.0–6.0)	5.0 (3.0–6.0)	-0.88*	0.18

Total time to complete the trail-making test (task A and task B), ratio of total trail-making task times, and total score for the reading span task (n = 25).

[a] Median (25%ile–75%ile)

[b,*] $p < 0.05$; TMT: trail-making task; RST: reading-span test.

https://doi.org/10.1371/journal.pone.0272035.t002

Table 3. Values for executive function before and after performing the sitting condition task.

		Before[a]	After[a]	Wilcoxon sum rank (z)[b]	Effect size (r)
TMT	Total time to complete task A (sec)	27.0 (23.5–30.0)	28.0 (21.5–30.5)	-0.80*	0.16
	Total time to complete task B (sec)	55.0 (44.5–66.5)	48.0 (40.0–57.5)	-1.79*	0.36
	Ratio for total times (BA)	2.2 (1.7–2.6)	1.8 (1.7–2.1)	-1.37*	0.27
RST	Total score of reading span task	4.0 (2.5–6.0)	5.0 (3.5–6.0)	-0.64*	0.13

Total time to complete the trail-making test (task A and task B), ratio of total trail-making task times, and total score for the reading span task (n = 25).

[a] Median (25%ile–75%ile)

[b,*] $p < 0.05$; TMT: trail-making task; RST: reading-span test.

https://doi.org/10.1371/journal.pone.0272035.t003

Table 4. Values for executive function before and after performing the mixed condition task.

		Before[a]	After[a]	Wilcoxon sum rank (z)[b]	Effect size (r)
TMT	Total time to complete task A (sec)	27.0 (22.0–32.5)	23.0 (20.5–28.5)	-2.34*	0.47
	Total time to complete task B (sec)	56.0 (44.5–65.5)	48.5 (38.0–60.8)	-1.77*	0.35
	Ratio for total times (BA)	2.1 (1.8–2.4)	1.8 (1.7–2.5)	-0.11*	0.02
RST	Total score of reading span task	4.0 (3.5–6.0)	4.0 (3.0–6.0)	-0.18*	0.04

Total time to complete the trail-making test (task A and task B), ratio of total trail-making task times, and total score for the reading span task (n = 25).

[a] Median (25%ile–75%ile)

[b,*] $p < 0.05$; TMT: trail-making task; RST: reading-span test.

https://doi.org/10.1371/journal.pone.0272035.t004

class as a whole to become more difficult to control [25], our findings indicate that the causes of such distractions are not related to stress resulting from using standing desks.

In relation to the findings for executive function, for the interference task of the Stroop test, a significant interaction was confirmed regarding the change in the number of correct answers (Fig 4). The number of correct answers in the interference task increased in conditions involving standing, and the 'inhibition' element of executive function apparently improved. A previous study examining the effect of body posture on the executive function of young university students reported that the total time required to complete the Stroop test was shorter when responses were given while standing [26]. Our study supports this and indicates that elementary school students can also improve their 'inhibition' by standing while working.

Mehta et al. [27] used functional near-infrared spectroscopy to perform brain imaging on high school students and found significant left frontal lobe activations during three of the five tasks for assessing executive function (the memory-span task, the trail-making test, and the

Stroop colour word test) after introducing standing desks for 27 weeks. In addition, Wick et al. [16], in an examination of 10–12-year-old children, administered two tasks for assessing 'inhibition' and 'updating' (the flanker task and digit span task, respectively) before and after the children used both a standing desk and a floor mat for 11 weeks. Wick et al. [16] reported that, although no significant difference was found, when compared to the control group, the intervention group showed a higher number of correct answers in the working memory task, and the effect size was larger than control group. According to the findings from these two previous studies, using a standing desk may improve not only 'inhibition', but also 'shifting' and 'updating'. However, in the present study, a significant change was only confirmed for the number of correct answers in the interference task of the Stroop test. It was not confirmed for the ratio of the total time to complete task A to that of task B in the trail-making test or the total score for the reading-span task (Tables 2–4). A possible reason for this result is that the measurement interval of this study was extremely short (45 minutes) when compared to those used in the previous studies (27 and 11 weeks, respectively). Conversely, Mazzoli et al. [28] analysed whether two school days' time spent sitting/stepping and the frequency of sit-to-stand transitions were associated with executive functions ('inhibition' and 'updating') improvements in schoolchildren. They reported that the child's sitting time and the number of sit-to-stand transitions were negatively associated with the response time and the number of correct answers for 'inhibition' (go/no-go task). However, no association with 'updating' was confirmed. Similarly, the present study using standing desks for only 45 minutes confirmed improved 'inhibition'. Although our findings did not definitely establish that using a standing desk for 45 minutes can improve all three sub-elements of executive function, we found that using a standing desk does affect children's 'inhibition'.

The present study investigated the effects of using standing desks for 45 minutes on stress levels and executive function of elementary school students. The findings showed that there was no difference in stress across the three conditions tested, and that the number of correct answers in the interference task of the Stroop test was higher for the standing condition and mixed condition as compared to the sitting condition. A strength of this study was the use of measures to clarify the biochemical effects of using standing desks for elementary school students for 45 minutes so as to parallel a single lesson period. This strength distinguished our study from previous ones that examined the effect of using a standing desk short-term (weeks), mid-term (months), and long-term (years). It was also the first study to investigate biochemical effects. However, there are several limitations to this research. First, the sample size was small; to confirm our findings it will be necessary to conduct a similar investigation in the future using a larger sample. Second, in this study, a relatively large number of subjects were unable to participate in all elements or had missing data. Moreover, the data from the Stroop tests of the subjects who participated in both experiments (2017, 2018) were analysed only for 2018. Caution is advised regarding the representative nature of the samples. Third, this study was conducted only on elementary school students; it is unclear whether similar results would be obtained in a study on junior high school or high school students. Fourth, this study was conducted in a laboratory, not in a school. It is unclear whether the effects observed in this study regarding the benefits of standing desks would persist in actual classroom situations.

Conclusions

Our study showed the effects of using standing desks on stress levels and executive function for an extremely short-term period representing one elementary school lesson: 45 minutes. Our findings indicated that using such desks while standing or alternating between standing

and sitting can improve the 'inhibition' element of executive function. No difference was found in the stress level between the conditions. These findings show that using standing desks for 45 minutes improved the 'inhibition' of executive function without excessively increasing stress levels.

Acknowledgments

The authors would particularly like to thank the participating children and their elementary school teachers and to thank research staffs.

Author Contributions

Conceptualization: Ryo Tanaka, Shingo Noi.

Data curation: Ryo Tanaka.

Formal analysis: Ryo Tanaka.

Funding acquisition: Shingo Noi.

Investigation: Ryo Tanaka.

Methodology: Ryo Tanaka, Shingo Noi.

Project administration: Shingo Noi.

Resources: Shingo Noi.

Validation: Ryo Tanaka, Shingo Noi.

Visualization: Ryo Tanaka.

Writing – original draft: Ryo Tanaka.

Writing – review & editing: Shingo Noi.

References

1. Tremblay MS, Aubert S, Barnes JD, Saunders TJ, Carson V, Latimer-Cheung AE, et al. Sedentary Behavior Research Network (SBRN)—terminology consensus project process and outcome. Int J Behav Nutr Phys Act. 2017; 14(75). https://doi.org/10.1186/s12966-017-0525-8 PMID: 28599680
2. Thivel D, Tremblay A, Genin PM, Panahi S, Rivière D, Duclos M. Physical activity, inactivity, and sedentary behaviors: definitions and implications in occupational health. Front Public Health. 2018; 6:288. https://doi.org/10.3389/fpubh.2018.00288 PMID: 30345266
3. World Health Organization. Physical activity fact sheet. 2018 [cited 9 April 2021]. Available from: https://www.who.int/health-topics/physical-activity.
4. Carson V, Hunter S, Kuzik N, Gray CE, Poitras VJ, Chaput J-P, et al. 2016. Systematic review of sedentary behaviour and health indicators in school-aged children and youth: An update. Appl Physiol Nutr Metab. 2016; 41(Suppl3):S240–S265. https://doi.org/10.1139/apnm-2015-0630
5. Tremblay MS, LeBlanc AG, Kho MC, Saunders TJ, Larouche R, Colley RC, et al. Systematic review of sedentary behaviour and health indicators in school-aged children and youth. Int J Behav Nutr Phys Activ. 2011; 8(98). https://doi.org/10.1186/1479-5868-8-98 PMID: 21936895
6. Russell V, Hotopf M. 2004. Childhood predictors of self-reported chronic fatigue syndrome/myalgic encephalomyelitis in adults: National birth cohort study. BMJ. 2004; 329(7472): 941. https://doi.org/10.1136/bmj.38258.507928.55 PMID: 15469945
7. Cardon G, Clercq D, De Bourdeaudhuij I, Breithecker D. Sitting habits in elementary schoolchildren: A traditional versus a 'moving school'. Patient Educ Couns. 2004; 54(2):133–142. https://doi.org/10.1016/S0738-3991(03)00215-5
8. Salmon J, Tremblay MS, Marshall SJ, Hume C. Health risks, correlates, and interventions to reduce sedentary behavior in young people. Am J Prev. 2011; 41(2):197–206. https://doi.org/10.1016/j.amepre.2011.05.001

9. Abbott RA, Straker LM, Mathiassen SE. Patterning of children's sedentary time at and away from school. Obesity. 2013; 21:E131–E133
10. Ridgers ND, Salmon J, Ridlet K, Connell EO, Arundell L, Timperio A. Agreement between activPAL and ActiGraph for assessing children's sedentary time. J Behav Nutr Phys Act. 2012; 9:5. https://doi.org/10.1186/1479-5868-9-15 PMID: 22340137
11. Howell W, Devereaux RS, Davis M, Collins J. 2000. Using the school environment to promote physical activity and healthy eating. Prev Med. 2000; 31:121–137. https://doi.org/10.1006/pmed.2000.0649
12. Mingen KE, Chao AM, Irwin ML, Owen N, Chorong Park, Whittemore R, et al. Classroom standing desks and sedentary behavior: A systematic review. Pediatrics. 2016; 137(2):e20153087. https://doi.org/10.1542/peds.2015-3087 PMID: 26801914
13. Clemes SA, Barber SE, Bingham DD, Ridgers ND, Fletcher E, Pearson N, et al. Reducing children's classroom sitting time using sit-to-stand desks: findings from pilot studies in UK and Australian primary schools. J Public Health. 2016; 38(3):526–533. https://doi.org/10.1093/pubmed/fdv084
14. Sherry AP, Pearson N, Clemes SA. 2016. The effects of standing desks within the school classroom: A systematic review. Prev Med Rep. 2016; 3: 38–347. https://doi.org/10.1016/j.pmedr.2016.03.016 PMID: 27419034
15. Kidokoro T, Shimizu Y, Edamoto K, Annear M. Classroom standing desks and time-series variation in sedentary behavior and physical activity among primary school children. Int J Environ Res Public Health. 2019; 16(11):1892. https://doi.org/10.3390/ijerph16111892 PMID: 31146330
16. Wick K, Faude O, Manes S, Zahner L, Donath L. I can stand learning: A controlled pilot intervention study on the effects of increased standing time on cognitive function in primary school children. Int J Environ Res Public Health. 2018; 15(2):356. https://doi.org/10.3390/ijerph15020356 PMID: 29462986
17. Ee J, Parry S, de Oliveira BIR, McVeigh JA, Howie E, Straker L. Does a classroom standing desk intervention modify standing and sitting behavior and musculoskeletal symptoms during school time and physical activity during waking time? Int J Environ Res Public Health. 2018; 15(8):1668. https://doi.org/10.3390/ijerph15081668
18. Ayala AMC, Salmon J, Timperio A, Sudholz B, Ridgers ND, Sethi P, et al. Impact of an 8-month trial using height-adjustable desks on children's classroom sitting patterns and markers of cardio-metabolic and musculoskeletal health. Int J Environ Res Public Health. 2016; 13(12):1227. https://doi.org/10.3390/ijerph13121227
19. Rhee JB, Benden ME. Stand-biased desk intervention on sleep quality of high school students: a pilot study using tri-axial accelerometery. Int J Environ Res Public Health. 2020; 17(1):37. https://doi.org/10.3390/ijerph17010037 PMID: 31861545
20. Parry S, de Oliveira BIR, McVeigh JA, Ee J, Jacques A, Straker L. Standing desks in a Grade 4 classroom over the full school year. Int J Environ Res Public Health. 2019; 16(19):3590. https://doi.org/10.3390/ijerph16193590 PMID: 31557874
21. Van den Berg V, Salimi R, De Groot RHM, Jolles J, Chinapaw MJM, Singh AS. 'It's a battle. . . you want to do it, but how will you get it done?': Teachers' and principals' perceptions of implementing additional physical activity in school for academic performance. Int J Environ Res Public Health. 2017; 14(10):1160. https://doi.org/10.3390/ijerph14101160 PMID: 28973967
22. Chatterton RT, Vogelsong KM, Lu Y-C, Ellman AB, Hudgens GA. Salivary alpha-amylase as a measure of endogenous adrenergic activity. Clin Physiol Funct Imaging. 1996; 16(4):443–448. https://doi.org/10.1111/j.1475-097X.1996.tb00731.x
23. Miyake A, Friedman NP, Emerson MJ, Witzki AH, Howerter A, Wager TD. The unity and diversity of executive function and their contributions to complex 'frontal lobe' tasks: A latent variable analysis. Cogn Psychol. 2000; 41(1):49–100. https://doi.org/10.1006/cogp.1999.0734
24. Higuchi K, Tomone T, Shinichi K, Rika I. The development of group reading span test and listening span test for children. J Fac Educ Shinshu Univ. 2001; 103:219–228. In Japanese.
25. Hinckson EA., Aminian S, Ikeda E, Stewart T, Oliver M, Duncan S, et al. Acceptability of standing workstations in elementary schools: A pilot study. Prev Med. 2012; 56(1):82–85. https://doi.org/10.1016/j.ypmed.2012.10.014
26. Rosenbaum D, Mama Y, Algom D. Stand by your stroop: Standing up enhances selective attention and cognitive control. Psychol Sci. 28 2017;(12):1864–1867. https://doi.org/10.1177/0956797617721270
27. Mehta RK., Shortz AE, Benden ME. Standing up for learning: A pilot intervention on the neurocognitive benefits of stand-biased school desk. Int J Environ Res Public Health. 2015; 13(1):59. https://doi.org/10.3390/ijerph13010059 PMID: 26703700
28. Mazzoli E, Teo W-P, Salmon J, Pesce C, He J, Ben-Soussan TD, et al. Associations of class-time sitting, stepping and sit-to stand transitions with cognitive functions and brain activity in children. Int J Environ Res Public Health. 2019; 16(9):1482. https://doi.org/10.3390/ijerph16091482 PMID: 31027380
